# Humanin Promotes Tumor Progression in Experimental Triple Negative Breast Cancer

**DOI:** 10.1038/s41598-020-65381-7

**Published:** 2020-05-22

**Authors:** Mariela A. Moreno Ayala, María Florencia Gottardo, Camila Florencia Zuccato, Matías Luis Pidre, Alejandro Javier Nicola Candia, Antonela Sofia Asad, Mercedes Imsen, Víctor Romanowski, Aldo Creton, Marina Isla Larrain, Adriana Seilicovich, Marianela Candolfi

**Affiliations:** 1 Instituto de Investigaciones Biomédicas (INBIOMED, UBA-CONICET), Facultad de Medicina, Universidad de Buenos Aires, Buenos Aires, Argentina; 2 Departamento de Biología Celular e Histología, Facultad de Medicina, Universidad de Buenos Aires, Buenos Aires, Argentina; 3 Instituto de Biotecnología y Biología Molecular (IBBM, UNLP-CONICET), Facultad de Ciencias Exactas, Universidad Nacional de La Plata, La Plata, Argentina; 4 Fundación Breast, Calle 7 432/479, B1902 La Plata, Argentina; 5 Hospital Italiano, Av. 51, B1900 La Plata, Argentina; 6 Centro de Investigaciones Inmunológicas Básicas y Aplicadas (CINIBA), Facultad de Ciencias Médicas, UNLP-CICPBA, La Plata, Argentina

**Keywords:** Breast cancer, Cancer models

## Abstract

Humanin (HN) is a mitochondrial-derived peptide with cytoprotective effect in many tissues. Administration of HN analogs has been proposed as therapeutic approach for degenerative diseases. Although HN has been shown to protect normal tissues from chemotherapy, its role in tumor pathogenesis is poorly understood. Here, we evaluated the effect of HN on the progression of experimental triple negative breast cancer (TNBC). The meta-analysis of transcriptomic data from The Cancer Genome Atlas indicated that HN and its receptors are expressed in breast cancer specimens. By immunohistochemistry we observed up-regulation of HN in TNBC biopsies when compared to mammary gland sections from healthy donors. Addition of exogenous HN protected TNBC cells from apoptotic stimuli whereas shRNA-mediated HN silencing reduced their viability and enhanced their chemo-sensitivity. Systemic administration of HN in TNBC-bearing mice reduced tumor apoptotic rate, impaired the antitumor and anti-metastatic effect of chemotherapy and stimulated tumor progression, accelerating tumor growth and development of spontaneous lung metastases. These findings suggest that HN may exert pro-tumoral effects and thus, caution should be taken when using exogenous HN to treat degenerative diseases. In addition, our study suggests that HN blockade could constitute a therapeutic strategy to improve the efficacy of chemotherapy in breast cancer.

## Introduction

Breast cancer is the most common cause of death by cancer in women^1^. Although new strategies have been developed for the treatment of breast tumors that express hormone receptors and/or human epidermal growth factor receptor 2 (Her2), there are no therapeutic options for patients with triple negative breast cancer (TNBC), for whom chemotherapy/radiotherapy remains the first-line treatment^2,3^. Since these tumors frequently develop chemo-resistance^4^, novel therapeutic targets are urgently needed to improve the treatment of TNBC.

A cytoprotective mitochondrial-derived peptide, humanin (HN), was discovered in healthy neurons of patients suffering Alzheimer’s disease^5^. HN was the first small open reading frame (ORF) identified within the mitochondrial DNA, encoded within the 16S rRNA gene (MT-RNR2)^6^. HN can be translated both in the mitochondrial matrix or the cytosol, resulting in biologically functional 21 and 24-amino acid peptides, respectively^7^. Small ORFs for six other small HN-like peptides (SHLPs) have been detected in the mitochondrial genome, two of which (SHLP2 and SHLP3) exhibit biological activity^8^. Thirteen MT-RNR2-like loci that encode for fifteen HN-like peptides were detected in the nuclear genome^9^. However, the expression of the mitochondrial gene MT-RNR2 is substantially higher than any nuclear isoform^9^.

HN regulates the mitochondrial apoptotic pathway by interaction with proteins of the Bcl-2 family^10^. Intracellular HN binds to proapoptotic proteins, such as Bax, tBid and BimEL inhibiting the release of cytochrome *c* and the apoptotic response to several cytotoxic stimuli^11–13^. HN can also be secreted, exerting autocrine, paracrine and endocrine effects upon interaction with membrane receptors. Two membrane receptors have been identified that bind circulating HN: (i) a trimeric receptor composed by the ciliary neurotrophic factor receptor (CNTFR), the IL27R (WSX-1) and the 130 kDa glycoprotein (gp130), which can trigger the activation of RAS/MAPKs, PI3K, JNK and STAT3; (ii) the formyl peptide receptor-like 1 (FPRL-1 or FPR2), which induces signal-regulated extracellular kinase activation (ERK 1/2)^10^. Activation of these receptors exerts cytoprotection in preclinical models of stroke, diabetes, Alzheimer’s disease, among other diseases^14^. In addition, it has been shown that cells can uptake exogenous HN, which rapidly localizes into the mitochondria where it blocks the formation of reactive oxygen species and restores mitochondrial bioenergetics, inhibiting cell senescence and death^15,16^.

HN exerts an antiapoptotic action in many different cell types, such as neurons, endothelial cells, pancreatic beta cells, germ cells and secretory cells of the anterior pituitary gland^10^. The cytoprotective role of HN has been described in different species, including humans, rats and mice^17–21^ and this peptide has been proposed to be a therapeutic target in many different diseases, such as Alzheimer’s disease, diabetes and atherosclerosis^10^. Although HN has been proposed to be a potential oncopeptide almost 2 decades ago^22^, its role in cancer development and treatment remains poorly understood. Since HN overexpression was detected in gastric cancer^23^, bladder tumor cells^24^, and pituitary tumor cells^13,18^, it was suggested that HN upregulation could play a role in tumorigenesis.

Although the cytoprotective effect of HN in normal cells exposed to chemotherapeutic drugs is well known^19,25^, its role in the response of tumor cells to cytotoxic drugs remains controversial. While it has been proposed that HN and its analogs may increase the sensitivity of tumor cells to bortezomib^26^ and cyclophosphamide^25^, HN has been shown to decrease apoptosis in glioma cells incubated with the glycosylation inhibitor tunicamycin^27^. Moreover, siRNA-mediated knock down of endogenous HN sensitized pituitary tumor cells^28^ and glioblastoma cells^12^ to proapoptotic stimuli. Inhibition of mitochondrial HN by intratumoral injection of baculoviral gene therapy vectors increased the expression of Bax and the apoptotic rate in the tumor and inhibited tumor growth, extending the survival of prolactinoma xenograft models^28^.

While the administration of HN and its analogs has shown promising results in preclinical models of degenerative diseases^10^, the controversy on the role of HN in cancer progression and chemoresistance needs to be addressed before translating these therapeutic approaches to the clinical practice. Thus, here we aimed to evaluate the expression and function of HN in human and murine breast tumor cells, as well as its role in tumor progression and chemoresistance in murine models of TNBC.

## Results

### Expression of HN in human and murine breast cancer cell lines and tissues

**Figure 1:**
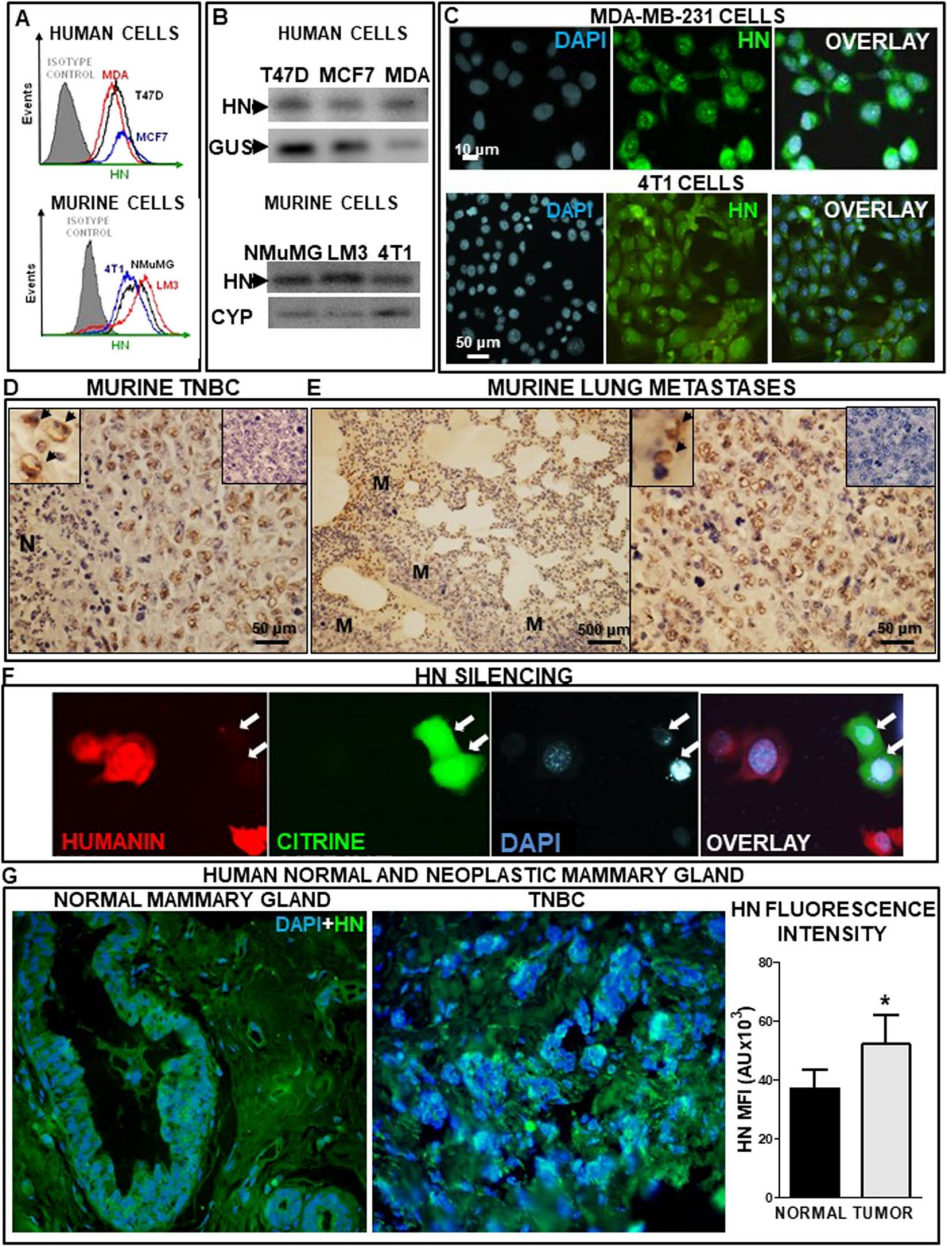
HN expression in human and murine breast tumor cells and tissues. HN expression was evaluated in human breast tumor cells MCF7, T47D and MDA-MB-231 (MDA) and in murine NMuMG, LM3 and 4T1 cells. (**A**) Representative histograms show the mean fluorescence intensity (MFI) of tumor cells, as assessed by flow cytometry. (**B**) Representative gel shows the expression of HN mRNA and internal control β-glucuronidase (GUS) or cyclophilin (CYP), as assessed by RT-PCR. (**C**) Images show the expression of HN in human (MDA-MB-231) and murine (4T1) TNBC cells, as evaluated by immunofluorescence. Images show HN (green), nuclei stained with DAPI (blue) and their overlay. (**D**) Representative microphotographs show HN expression as assessed by immunohistochemistry in paraffin sections of primary 4T1 TNBC tumors. Insets show cells expressing HN (left, arrows) and a control without primary antibody (right). N: necrotic area. (**E**) Low (left) and high (right) magnification images showing HN expression in metastatic nodules (M) in lungs from 4T1 TNBC-bearing mice. Insets show cells expressing HN (left, arrows) and a control without primary antibody (right). (**F**) Images show the expression of HN in murine (4T1) TNBC cells transfected with p.shHN, as evaluated by immunofluorescence. Images show HN-positive cells (red), citrine-positive (transfected cells, green), nuclei stained with DAPI (blue) and their overlay. Arrows indicate transfected cells that do not express HN. (**G**) Representative microphotographs show HN expression (green) as assessed by immunofluorescence in paraffin sections from mammary gland samples from healthy donors that underwent cosmetic breast surgery (left panel) and tumor biopsies from TNBC patients (right panel). Nuclei stained with DAPI (blue). The graph depicts the median fluorescence intensity (MFI) of HN immunofluorescence in samples of normal mammary gland (n = 5) or biopsies from breast tumor patients (n = 5), as analyzed with ImageJ software. To obtain the fluorescence intensity/field for each sample a mean value was calculated by assessing 20 fields.

Since the expression of HN has not been evaluated in breast cancer cells before, we first assessed the presence of HN and its mRNA in human and murine breast tumor cell lines. We detected HN in human MCF7 and T47D luminal breast tumor cells and MDA-MB-231 TNBC cells, as assessed by flow cytometry (Fig. 1A). Similar findings were observed in murine breast cell lines. We found expression of HN in HER2^+^ LM3 cells and TNBC 4T1 cells, as well as in non-tumorigenic breast epithelial cells NMuMG (Fig. 1A). In addition, HN expression was detected by RT-PCR in all human and murine cell lines evaluated (Fig. 1B). Expression of HN was confirmed by immunofluorescence in TNBC cells (Fig. 1C). We also evaluated HN expression in an *in vivo* TNBC murine model. 4T1 cells were injected in the flank of syngeneic Balb/c mice and HN immunohistochemistry was performed in paraffine sections from the primary tumor (Fig. 1D, Suppl. Figure 1) and the lung metastases that spontaneously develop in these mice (Fig. 1E, Suppl. Figure 1). We found profuse HN expression in 4T1 primary tumors (Fig. 1D) and lung metastases (Fig. 1E). In order to assess the specificity of HN staining, we transfected 4T1 TNBC cells with a plasmid encoding a shRNA specific for murine HN (p.shHN), which was constructed to assess the effect of HN silencing in these cells. In order to readily detect transfected cells this plasmid also encoded for the green fluorescent protein citrine. As expected, the expression of HN was undetectable in 4T1 TNBC cells that were effectively transfected with the plasmid and, thus, showed green fluorescence (Fig. 1F), which indicates that HN staining is specific.

We next evaluated the expression of HN by immunofluorescence in sections of normal human mammary gland obtained from women undergoing cosmetic breast reduction, as well as in biopsies from TNBC patients. While HN expression was detected in cells of the normal breast, HN expression was stronger and more spread in TNBC biopsies (Fig. 1G, Supp. Figure 2). Quantification of the median fluorescence intensity in each sample (n = 5 per group) indicated that HN expression is upregulated in breast cancer biopsies (Fig. 1G).

### Effect of HN on the response of murine and human TNBC cells to cytotoxic stimuli

**Figure 2:**
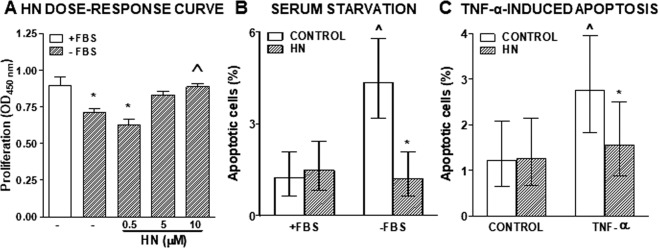
Effect of HN on proliferation and apoptosis of murine TNBC cells. (**A**) 4T1 cells were incubated with or without FBS in the presence of different concentrations of HN for 18 h (n = 4 replicates/condition) and proliferation was assessed by BrdU incorporation (ELISA). *p < 0.05 vs. +FBS, ^p < 0.05 vs.-FBS (ANOVA followed by Tukey´s test). (**B**) 4T1 cells were incubated with or without FBS in the presence of HN (10 μM) for 24 h and apoptosis was assessed by the TUNEL method. Bars indicate the percentage of apoptotic cells ± 95% confidence limits (CL) of the total number of cells counted in each specific condition (n ≥ 1000 cells/group). Confidence intervals for proportions were analyzed by the χ^2^ test: ^p < 0.05 vs. respective control +FBS, *p < 0.05 vs. respective control without HN (χ^2^ test). (**C**) 4T1 cells were incubated in medium with FBS in the presence of HN (10 μM) for 2 h before addition of the proapoptotic cytokine TNF-α (10 ng/ml) for further 24 h. Apoptosis was assessed by the TUNEL method. Bars indicate the percentage of apoptotic cells ± 95% confidence limits (CL) of the total number of cells counted in each specific condition (n ≥ 1000 cells/group). Confidence intervals for proportions were analyzed by the χ^2^ test: ^p < 0.05 vs respective control without TNF-α, *p < 0.05 vs respective control without HN (χ^2^ test).

**Figure 3:**
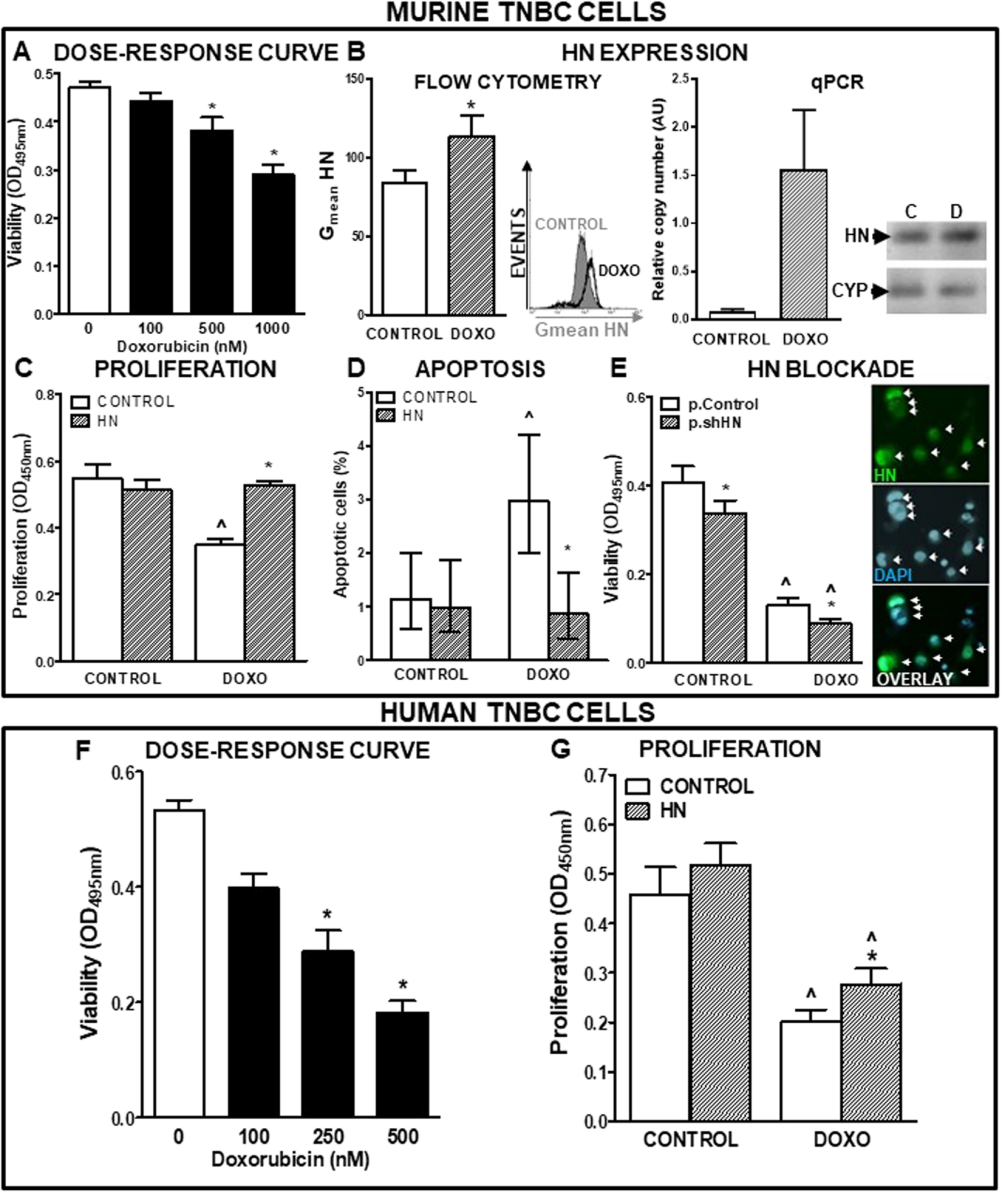
Effect of HN on the chemosensitivity of TNBC cells. (**A**) Murine TNBC 4T1 cells were incubated in medium with FBS with liposomal Doxorubicin at different concentrations for 72 h (n = 6 replicates/condition). Viability was assessed by MTT assay. *p < 0.05 vs. control without Doxorubicin. ANOVA followed by Tukey’s test. (**B**) Mean fluorescence intensity (MFI) of HN in 4T1 cells as evaluated by flow cytometry (left panel) (n = 3 replicates/condition) and qPCR (right panel) following 24 h incubation with Doxorubicin (DOXO, 500 nM). Representative histogram and qPCR products are shown. *p < 0.05 Student’s *t* test. (**C**) 4T1 cells were incubated in medium with FBS with HN (10 μM) for 2 h before adding DOXO (500 nM) for further 72 h (n = 5 replicates/condition). Proliferation was assessed by BrdU incorporation (ELISA). ^p < 0.05 vs. respective control without DOXO; *p < 0.05 vs. respective control without HN. ANOVA followed by Tukey´s test. (**D**) 4T1 cells were incubated in medium with FBS with HN (10 μM) for 2 h before adding DOXO (500 nM) for further 24 h and apoptosis was evaluated by the TUNEL method. Bars indicate the percentage of apoptotic cells ± 95% confidence limits (CL) of the total number of cells counted in each specific condition (n ≥ 1000 cells/group). Confidence intervals for proportions were analyzed by the χ^2^ test: ^p < 0.05 vs. respective control without DOXO; *p < 0.05 vs. respective control without HN. χ^2^test. (**E**) 4T1 cells were transfected with p.Control or p.shHN and incubated with DOXO (500 nM) for 72 h (n = 6 replicates/condition). Viability was assessed by MTT assay. *p < 0.05 vs. p.Control, ^p < 0.05 vs. control without DOXO. ANOVA followed by Tukey´s test. *Inset:* Representative microphotograph showing positive cells for citrine (transfected cells, green, upper panel), nuclei stained with DAPI (middle panel) and their overlay (lower panel). Arrows indicate transfected cells. (**F**) MDA-MB 231 cells were incubated in medium with FBS containing liposomal Doxorubicin (DOXO) at different concentrations for 72 h (n = 6 replicates/condition). Viability was assessed by MTT assay. *p < 0.05 vs. control without DOXO. ANOVA followed by Tukey´s test. (**G**) MDA-MB 231 cells were incubated in medium with FBS and HN (10 μM) for 2 h before adding DOXO (250 nM) for further 72 h (n = 5 replicates/condition). Proliferation was assessed by BrdU incorporation (ELISA). ^p < 0.05 vs. respective control without DOXO, *p < 0.05 vs. respective control without HN. ANOVA followed by Tukey’s test.

Considering that we have previously reported an anti-apoptotic role of HN in normal and tumor anterior pituitary cells^13,18^, we evaluated whether HN modulates the response of TNBC cells to cytotoxic stimuli. We first assessed the effect of different doses of exogenous HN on the proliferation of 4T1 murine TNBC cells incubated in absence of serum (Fig. 2A). Serum starvation inhibited the proliferation of 4T1 cells. While low doses of HN (0.5 and 5 µM) did not significantly affect the proliferation of serum-deprived cells, 10 µM HN reverted the anti-proliferative effect of serum deprivation and restored the proliferation rate of 4T1 cells to control levels. In view of these findings, 10 µM was the concentration chosen for all the following experiments. Then, we explored whether HN modulates the apoptotic response of 4T1 cells to cytotoxic stimuli, such as serum starvation (Fig. 2B) and the proapoptotic cytokine TNF-α (Fig. 2C). Apoptotic cells were identified by TUNEL 24 h after stimulation. HN did not affect the basal apoptotic rate of these cells. Both stimuli increased the percentage of TUNEL-positive cells, an effect that was reverted by the addition of HN. To further investigate the effect of HN in the apoptotic response of TNBC cells, we analyzed whether HN could affect the sensitivity of 4T1 cells to chemotherapy. We incubated 4T1 cells in the presence of different doses of liposomal doxorubicin (DOXO, Fig. 3A) and evaluated cell viability by MTT assay. While 4T1 cells were resistant to 100 nM DOXO, higher doses decreased cell viability in these cell cultures. We then evaluated whether cellular stressors could modulate the expression of HN. While serum deprivation did not significantly modify the expression of HN in 4T1 cells (data not shown), treatment with DOXO (500 nM) upregulated it, as assessed by flow cytometry and qPCR (Fig. 3B). We next assessed the effect of HN on the proliferation (Fig. 3C) and apoptosis (Fig. 3D) of 4T1 cells incubated with 500 nM DOXO. HN impaired both the anti-proliferative and pro-apoptotic effects of DOXO in 4T1 cells. To evaluate the role of endogenous HN in the response of 4T1 cells to DOXO, we transfected the cells with p.shHN, which exhibited very good transduction efficiency. HN silencing decreased the viability of 4T1 cells and enhanced the cytotoxic effect of DOXO (Fig. 3E).

We also assessed the chemo-sensitivity of human TNBC MDA-MB-231 cells, which were incubated in the presence of different concentrations of DOXO (Fig. 3F). Doses of 250 nM and above reduced the viability of MDA-MB-231 cells. When these cells were incubated in the presence of 250 nM DOXO, we detected an anti-proliferative effect that was partially reverted by concomitant incubation with HN (Fig. 3G).

### Effect of HN on the progression and chemosensitivity of experimental TNBC

**Figure 4:**
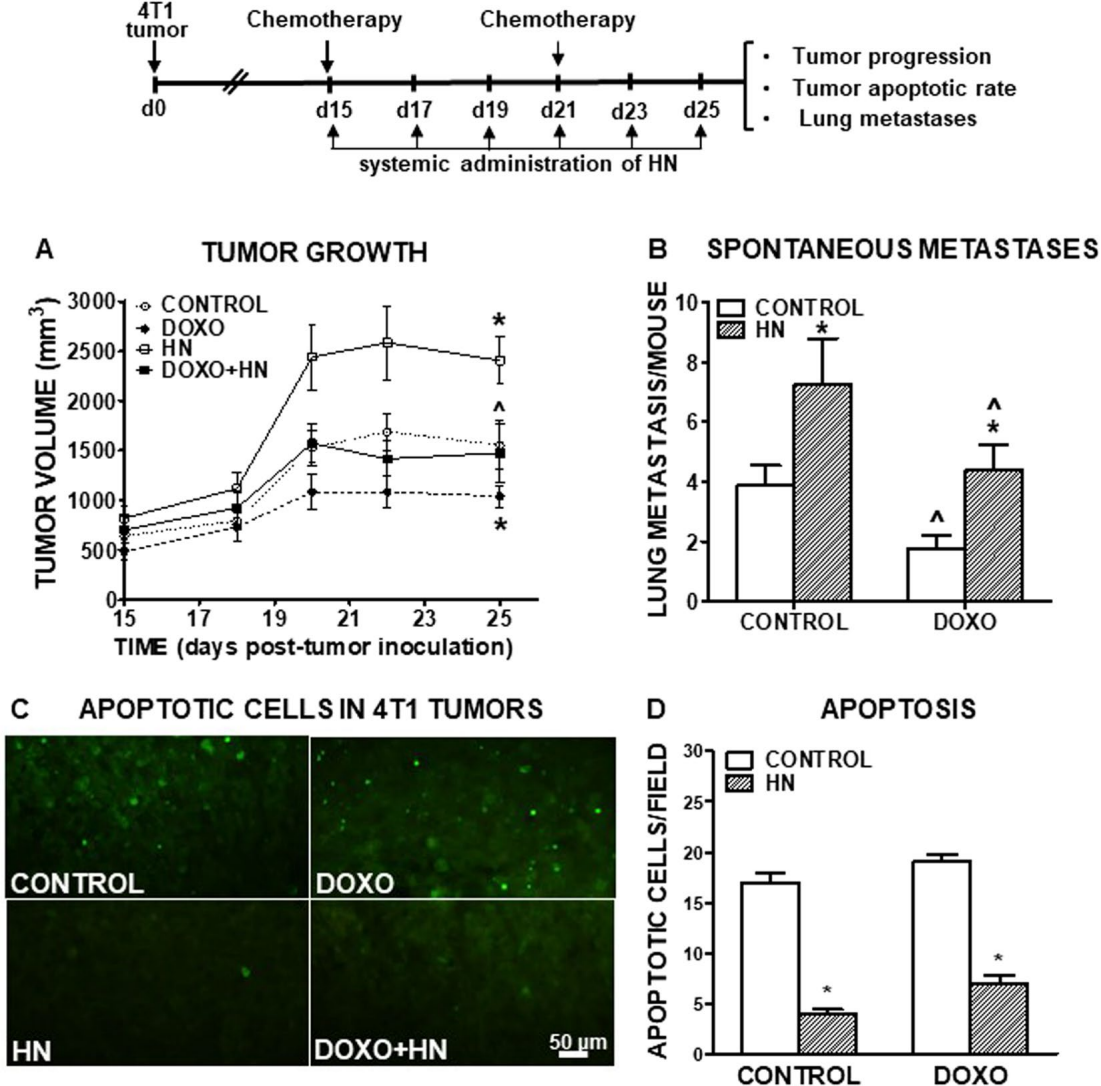
Effect of HN on the progression and chemosensitivity of experimental TNBC. BALB/c mice were s.c. inoculated with 3 × 10^5^ murine TNBC 4T1 cells and treated with an injection of liposomal Doxorubicin (DOXO, 100 µg/mouse) at days 15 and 21 post-tumor inoculation. Mice received 6 injections of HN (10 μg/mouse) administered every other day starting at day 15. (**A**) Tumor size was measured with caliper 3 times a week. *p < 0.05 DOXO vs. control; ^p < 0.05 DOXO vs. DOXO + HN. Multiple regression analysis (n = 8). (**B**) The number of spontaneous lung metastases was evaluated at day 25 post-tumor inoculation. ^p < 0.05 vs. respective control without DOXO; *p < 0.05 vs. respective control without HN. ANOVA followed by Tukey´s test. (**C**) Representative microphotographs show apoptotic cells in 4T1 tumor sections, as assessed by the TUNEL method. (**D**) Apoptotic cells/field. *p < 0.05 vs. respective control without DOXO. ANOVA followed by Tukey’s test.

In order to assess the effect of HN on the progression and chemosensitivity of TNBC *in vivo* we used an experimental murine model. BALB/c mice were s.c. inoculated with 4T1 murine TNBC cells. When tumors were macroscopic (~day 15), mice were treated with an i.p. injection of DOXO on days 15 and 21. Mice also received 6 i.p. injections of HN every other day, starting on day 15. Treatment with DOXO exerted a mild inhibitory effect on tumor growth that was impaired by the concomitant administration of HN (Fig. 4A, Suppl. Figure 3). Interestingly, administration of HN alone significantly accelerated tumor growth when compared to control mice. We determined the apoptotic rate in tumor sections from each experimental group by the TUNEL method and the number of spontaneous metastases developed in the lung of tumor-bearing mice at the end of the treatment (Fig. 4B–D). HN reduced the number of apoptotic cells in tumors from both control and DOXO-treated mice (Fig. 4C–D). We found that DOXO exerted an anti-metastatic effect that was abrogated when mice received simultaneous administration of HN (Fig. 4B). Of note, the administration of HN alone significantly promoted the development of spontaneous lung metastases when compared to control mice.

### Expression of HN and its receptors in human breast cancer

The expression of HN mRNA was evaluated by bioinformatics analysis of transcriptomic data from different bioprojects available in NCBI. HN expression was found in all samples reported, which included normal and tumor breast cells, luminal and basal cell lines, as well as primary breast tumors of all types and their metastases (Table 1).

**Table 1:** Human breast cancer cell lines, normal and neoplastic tissues in which HN mRNA is detected.

SOURCE	TISSUE TYPE			SAMPLE SIZE	PLATFORM	ACCESSION NUMBER
	PRIMARY TUMOR TYPE		METASTASIS			
CELL LINES	BASAL	BT-549		n = 1/cell line	Illumina HiSeq 4000	PRJNA434599
		HBL-100				
		SUM-159				
	LUMINAL	BT-474				
		T47D				
		MCF7				
MAMOPLASTY REDUCTION	NORMAL ADULT BREAST EPITHELIUM			n = 3 donors	Illumina HiSeq 2500	PRJNA450412
PATIENT-DERIVED XENOGRAFT	TNBC			n = 11 patients	Illumina HiSeq 2500	PRJNA451206
	BASAL-LIKE HER2 +			n = 1 patient		
	LUMINAL B ER +			n = 8 patients		
	LUMINAL B HER +			n = 1 patient		
	ER + /PR-			n = 1 patient		
	TNBC		BRAIN METASTASIS	n = 2 patients	Illumina HiSeq 2500	PRJNA434130
PRIMARY TUMOR	ER + TUMOR			n = 12 patients	Illumina HiSeq 2500	PRJNA327871
	TUMORSPHERE					
	TNBC			n = 6 patients	Illumina HiSeq 2500	PRJNA305054
	HER2 +			n = 2 patient		
	ER + /PR +			n = 2 patients		
	LUMINAL B HER +			n = 1 patient		
METASTASES			LUNG	n = 3 patients	Illumina HiSeq 2500	PRJNA377764
			PLEURA	n = 14 patients		
			LYMPH NODE	n = 15 patients		
			SKIN	n = 5 patients		
			LIVER	n = 6 patients		
			OVARY	n = 2 patients		
			CHEST WALL	n = 3 patients		

**Figure 5:**
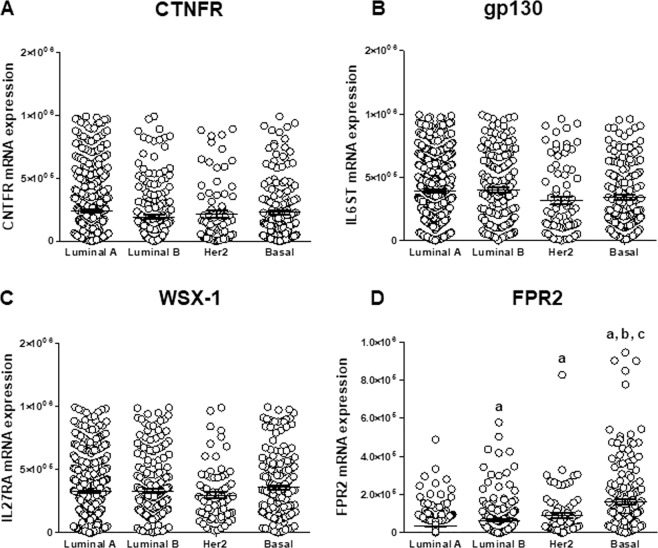
Bioinformatic analysis of expression of the trimeric receptor subunits and FPR2 in breast cancer specimens. Expression levels of HN trimeric receptor subunits (**A**) CNTFR (CNTFR mRNA), (**B**) gp130 (IL6ST mRNA), (**C**) WXS-1 (IL27RA mRNA) and (**D**) FPR2 (FPR2 mRNA) were obtained from breast cancer data deposited at the cBioportal for cancer genomics (TCGA PanCancer Atlas). Luminal A n = 461, Luminal B n = 339. HER2 n = 78, Basal n = 169. **a**: p < 0.05 vs. Luminal A; **b**: p < 0.05 vs Luminal B, **c**: p < 0.05 vs. Her2. Oneway ANOVA followed by Tukey’s test.

Considering that endogenous and exogenously administered HN can interact with membrane receptors, we performed the bioinformatic analysis of transcriptomic data from human specimens of breast cancer (TCGA). While the expression of the HN trimeric receptor subunits was similar in all subtypes of breast tumors (Fig. 5A–C), the expression of FPR2 differs with tumor type, being highest in TNBC (Fig. 5D).

## Discussion

Since its discovery in early 2000s HN has been proposed as a potential oncopeptide^22^. However, its role in the apoptotic response of tumor cells and in tumor progression has been barely explored in tumor models. Our study shows that HN facilitates tumor progression and chemo-resistance in an experimental model of breast cancer. HN has been shown to protect several cell types from apoptosis, including neurons, endothelial cells, and pancreatic β-cells^10^. In the context of cancer, the role of HN is poorly understood. HN is overexpressed in biopsies from patients with gastric^23^ and bladder cancer^24^ when compared to non-neoplastic surrounding tissue. We have previously shown that HN is overexpressed in rat pituitary tumor cells, when compared to normal pituitary cells^18^. Here, we detected expression of HN in primary tumor and metastases of mice bearing experimental TNBC. Our bioinformatics analysis of transcriptomic databases revealed expression of HN mRNA in normal breast tissue and breast tumors specimens of all types, as well as in their metastases. We also detected HN protein expression in both normal and neoplastic breast tissue, being more intense and wide-spread in the latter. Thus, it is possible that HN could play a role in the carcinogenic process. In fact, our analysis of transcriptomic data from breast tumor specimens indicates that HN receptors are present in breast tumors of all types, and that FPR2 expression is highest in TNBC. It is possible that the interaction of locally produced or circulating HN with FPR2 could be involved in the pathogenesis of TNBC. HN is considered a mitokine, *i.e*. a mitochondrial-derived signal that is released in response to mitochondrial stress and signals to distant tissues^14^. Circulating HN has been detected in patients with cutaneous T-cell lymphoma, but not in healthy donors^29^. In addition, lower levels of circulating HN were detected in patients with coronary endothelial dysfunction when compared to those with preserved coronary endothelial function^30^, suggesting that circulating HN could hold value as a biomarker in cancer and other diseases.

Although HN has been shown to be cytoprotective in normal cells, such as male germ cells and leukocytes exposed to chemotherapeutic drugs^25,31^, the role of this peptide in the chemosensitivity of tumor cells remains controversial. It has been shown that HNG, a potent synthetic HN analog in which the serine at position 14 has been replaced by glycine, protects bone cells from the cytotoxic effect of proteasome inhibitor bortezomib, while improving the apoptotic response of neuroblastoma and medulloblastoma in human xenograft models^26^. However, the mechanism by which this HN analog decreases tumor angiogenesis and increases tumor cell apoptosis is not clear in that report. The HN analog has also been shown not only to protect leukocytes from cyclophosphamide, but to boost its anti-metastatic effect in melanoma models^31^. Our findings using HN contradict this report as HN administration impaired the anti-metastatic effect of chemotherapy and clearly stimulated the development of spontaneous lung metastases *per se* in TNBC tumor-bearing mice, and impaired the anti-metastatic effect of chemotherapy with doxorubicin. The discrepancies between previous reports and our present results could be related to the different doses used, the use of HN analogs vs. wild type HN, the utilization of chemotherapeutic drugs with different mechanism of action, the tumor cell types studied, as well as the tumor models used, i.e. the i.v. injection of tumor cells in Ref. ^31^ vs. our s.c. TNBC model that develops spontaneous lung metastases^32^, or the use of immunosuppressed hosts^26,33^ vs. our immune-competent mouse model. Since the analog HNG has shown higher affinity by membrane HN receptors and a more potent antiapoptotic effect than HN in several cell types^34^, it is possible that *in vivo* HN and HNG could interact differently with the receptors present in tumor and stromal cells. On the other hand, it has been previously described that the protective effect of HN depends on the cell types and the cytotoxic stimuli applied. Although HN inhibits the cytotoxic effect of Prion-peptide_118–135_ in neurons, it fails to protect them from Prion peptide_106–126_, effects that have been related to the different mechanism of action of these peptides^35^. In addition, although the cytoprotective effect of HN has been extensively demonstrated in neurons, this peptide does not protect them from some cytotoxic stimuli, including etoposide and Fas^36^. Considering that the administration of HN and its analogs have been proposed for the treatment of several diseases^10^, the controversy over the role of exogenously administrated HN needs to be elucidated before being translated to the clinic.

Although the regulation of HN expression remains largely unknown, mitochondrial microRNAs (hsa-miR-mit3 and hsa-miR-mit4) have been proposed to bind the MT-RNR2 gene, which encodes HN and 16SrRNA^37^. However, their role in the regulation of HN expression remains to be determined. Our previous results indicate that steroid hormones modulate the expression of HN in pituitary cells^18^. HN expression is higher in male than female pituitary gland and estradiol exerts a direct inhibitory action on HN expression in pituitary cells^18^. The expression of HN is increased by cellular stressors, such as oxidative stress in skeletal muscle cells^38^, as well as hypoxia and serum starvation in human extravillous trophoblast cells^39^. While serum deprivation did not affect HN expression in TNBC cells, we found that chemotherapy with DOXO upregulated its expression. Trim11 also modulates the expression of HN, as it binds and destabilizes intracellular HN^40^. Thus, a dysregulation of this pathway could be involved in of HN overexpression in cancer.

As far as we know, our study shows for the first time that HN is present and exerts a cytoprotective effect on breast cancer cells. We demonstrate here that HN impairs the pro-apoptotic and anti-proliferative effects of different insults such as serum deprivation, TNF-α treatment and chemotherapy. HN is secreted and binds with cell surface receptors but also acts intracellularly through the interaction with Bax and other proteins of the Bcl-2 family^10^. We have previously reported that the cytoprotective effect of HN in pituitary tumor cells involves STAT3 activation and inhibition of Bax translocation to mitochondria^13^. The inhibition of intracellular HN using a gene therapy vector that encodes a short-hairpin RNA targeting HN upregulates the expression of Bax and exerts antitumor effects in a rat prolactinoma model^28^. Tallying with these observations, we found that HN silencing using a mouse HN-specific shRNA also exerted a cytotoxic effect in TNBC cells and sensitized them to chemotherapy, suggesting that HN expression may play a role in the resistance of TNBC to chemotherapy. In fact, our study indicates that HN is upregulated in biopsies of TNBC patients when compared to normal mammary gland samples and the meta-analysis of transcriptomic data shows that the HN receptor FPR2 is substantially upregulated in TNBC.

In summary, we found that HN and its receptors are upregulated in TNBC, and that exogenous and endogenous HN protects these cells from several cytotoxic and anti-proliferative stimuli. In addition, administration of HN facilitates tumor progression and chemo-resistance in TNBC models, and, thus, caution should be taken when using HN analogs to treat degenerative diseases. Our study suggests that HN could constitute a novel therapeutic target to improve the efficacy of chemotherapy in TNBC.

## Materials and Methods

### Patients and datasets

HN expression was assessed in paraffin sections obtained from TNBC patients that underwent tumor resection (n = 5) at Hospital Italiano, La Plata and from healthy donors that underwent breast cosmetic surgery (n = 5) at Fundación Breast by Dr. Aldo Creton. The study was conducted with the approval of and in accordance with the relevant guidelines and regulations of the Bioethics and Research Ethics Committee (Protocol N° 41 /2018 COBIMED). Written informed consent was obtained from all patients that participated in the study. Inclusion criteria included the ability to give informed consent and age above 18 years old.

Expression levels of HN trimeric receptor subunits and FPRL-1 (FPR2) were obtained from breast cancer data deposited at the cBioportal for cancer genomics (TCGA PanCancer Atlas). The number of patients was as follows: Luminal A n = 461, Luminal B n = 339. HER2 n = 78, Basal n = 169. Transcriptomic data of HN expression in human breast tumor specimens and cell lines were obtained at The Cancer Genome Atlas (TCGA). The number of samples is indicated in Table 1.

### Drugs

Humanin peptide was obtained from Genemed Synthesis (San Antonio, TX). Culture media were obtained from Gibco (Invitrogen, Carlsbad, CA), fetal bovine serum (FBS) from Natocor (Buenos Aires, Argentina) and all terminal deoxynucleotidyltransferase-mediated dUTP nick end-labeling (TUNEL) reagents from Roche Molecular Biochemicals (Mannheim, Germany). Liposomal doxorubicin hydrochloride (DOXO) was obtained from Dalmonar^®^, Tuteur (Argentina), and anti-rabbit IgG and anti-rabbit fluorescein-conjugated secondary antibody from Vector Laboratories Inc. (Burlingame, CA). Primers were obtained from Macrogen (Rockville, MD), Biodynamics (GenScript Inc, Piscataway, NJ) and Integrated DNA Technologies (Buenos Aires, Argentina) and the materials indicated below.

#### Cell Lines

The HER2^+^ LM3 cell line, developed from a murine mammary adenocarcinoma that spontaneously arose in a BALB/c mouse at the animal care facility of the Instituto de Oncología “Angel H. Roffo” (Buenos Aires, Argentina)^24^ was kindly provided by Dr. Elisa Bal de Kier Joffe. A highly metastatic murine TNBC cell line (4T1) derived from a spontaneous mammary tumor in a BALB/c mouse (ATCC, Cat# CRL-2539), was kindly provided by Dr. Osvaldo Podhajcer from Fundación Instituto Leloir, Buenos Aires, Argentina. Murine non-neoplastic mammary epithelial cells NMuMG (ATCC, Cat# CRL-1636), as well as luminal A human cell lines MCF7 (ATCC, Cat# HTB-22) and T47D (ATCC, Cat# HTB-133), and the human TNBC cell line MDA-MB-231 (ATCC, Cat# HTB-26), were kindly provided by Dr. Andrea Randi (Departamento de Bioquímica Humana, Facultad de Medicina, Universidad de Buenos Aires). Cells were cultured in media (LM3 and 4T1: RPMI; NMuMG: MEM; MCF7, T47D and MDA-MB-231: DMEM) containing 10% FBS, 1% L-glutamine (GIBCO) and 100 IU/ml penicillin (Invitrogen).

### Immunohistochemistry

HN expression was assessed by immunocytochemistry using anti-HN antibodies from Sigma (1:100, cat# H2414) and Novus Biol (1:100, cat# NB300-246), as we previously described^18^ HN expression in paraffin sections from TNBC patients that underwent tumor resection and from healthy donors that underwent breast cosmetic surgery was quantified by fluorescence intensity using ImageJ software (Version: 1.52t). (see Supplementary Information).

### HN detection by Flow cytometry

The expression of HN was assessed in tumor cells using anti-HN antibody (1 μg/μl, Sigma cat# H2414) followed by anti-rabbit antibody conjugated with FITC (Vector, 1:50) using a FACScan (Becton Dickinson, San Jose, CA) as previously described (^13^, see Supplementary Materials).

### RNA isolation, RT-PCR and qRT-PCR

RNA from murine and human cells was extracted using Trizol reagent (Invitrogen) according to the manufacturer’s protocol. One µg of total RNA was reverse-transcribed using SuperScript II Reverse Transcriptase according to the manufacturer’s protocol (Invitrogen). For RT-PCR, amplification of HN cDNA was performed using Taq DNA polymerase (Invitrogen) in a thermal cycler (UNO II Biometra, Göttingen, Germany) using the following primers: mHN: forward 5′-TGGCTAAAGGAGGGTTCAACTG-3′; reverse 5′-AGAAAACCAAGGGTCTTCTCGTC-3′; hHN: forward 5′-TGTCAACCCAACACAGGCATG-3′; reverse 5′-AAACAGGCGGGGTAAGATTTG-3′. Human β-glucuronidase (hGUSB, forward 5′-CCTGCGTCCCACCTAGAATC-3′; reverse 5′-ATACGGAGCCCCCTTGTCT-3′) and murine cyclophilin (mCYP, forward 5′-TATCTGCACTGCCAAGACTGAGTG-3′; reverse 5′-CTTCTTGCTGGTCTTGCCATTCC-3′) were used as internal controls. Murine HN expression was also assessed by qRT-PCR, using the mCYP as a reference mRNA, and performed as previously described^28^ (see Supplementary Information).

### BrdU incorporation assay

4T1 cells were incubated with or without FBS in the presence of 0.5, 1 or 10 μM HN for 18 h or in medium with FBS containing HN (10 μM) for 2 h before adding DOXO (500 nM) for further 72 h. MDA-MB-231 cells were incubated with in medium with FBS containing HN (10 μM) for 2 h before adding DOXO (250 nM) for further 72 h. Proliferation was assessed by BrdU incorporation as previously described (Supplementary Information)^32^.

### Cell viability assay (MTT)

4T1 and MDA-MB-231 cells were incubated in medium with FBS with different doses of DOXO for 72 h and cell viability was assessed as previously described (Supplementary Information)^18,32^.

### Microscopic detection of DNA fragmentation by TUNEL

The apoptotic response of 4T1 cells was evaluated after incubation with or without serum and HN (10 μM) for 24 h. In other experiments, 4T1 cells were incubated in medium with FBS containing HN (10 μM) for 2 h before the addition of TNF-α (50 ng/ml) or DOXO (500 nM) for further 24 h. Apoptotic cells were detected using the TUNEL method as we previously described^13,18^ (Supplementary Information).

### Plasmid construction and transfections

The shRNA-coding dsDNA comprising the mHN RNA sequence (shRNA-mHN:CCTTTCAGTGAAGAGGCTGAAAAGTTCTCTTTCAGCCTCTTCACTGAAAGGTTTTTT) was synthesized fused to the U6 promoter and cloned in a bicistronic pUC57 vector (p.shHN) as previously described^28^. In order to detect transfected cells, the construct included the coding sequence for citrine fluorescent reporter protein under the control of the cytomegalovirus (CMV) major immediate-early (IE) promoter.

4T1 cells were transfected with 1 μg of p.shHN or control plasmid DNA using Lipofectamine 2000 (Invitrogen, Carlsbad, CA) and incubated for 24 h. Then, cells were fixed to assess transduction efficiency or to evaluate HN expression, or incubated with DOXO (500 nM) for further 72 h to measure cell viability.

### TNBC tumor model

Female adult BALB/c (6–8 weeks old) were purchased at the vivarium of Facultad de Ciencias Veterinarias, Universidad Nacional de La Plata, and kept under regulated conditions of light (12 h light-dark cycles) and temperature (20–25 °C). Mice were fed with standard lab chow and water *ad libitum*. All animal procedures were conducted according to the NIH guidelines and approved by the Institutional Ethical Committee, Facultad de Medicina, Universidad de Buenos Aires.

BALB/c female mice were inoculated s.c. into the flank with 3 × 10^5^ 4T1 cells as previously described^32^. When tumors were macroscopic (day 15), mice were treated with an i.p. injection of DOXO (100 µg/mouse), that was repeated on day 21. Mice were also treated with 6 injections of HN (10 μg/mouse), administered every other day, starting on day 15. Tumor measurement was performed 3 times per week using a caliper. Tumor volume was calculated with the following formula: (width^2^xlength)/2. After treatment, mice were euthanized under deep anesthesia by terminal perfusion with Tyrode solution, followed by Bouin fixative solution. Lungs were dissected and spontaneous metastases were counted under binocular microscope. Tumors were dissected and processed for TUNEL staining.

### Statistical analysis

Data were graphed and analyzed using GraphPad Prism version 5.00 software (GraphPad Software). Tumor growth was analyzed by multiple regression analysis. Differences in the expression of HN receptor mRNA, number of lung metastases, BrdU incorporation and MTT data were analyzed by analysis of variance (ANOVA) followed by Tukey´s test. The number of apoptotic cells evaluated by TUNEL in slides from three independent experiments was expressed as percentage of TUNEL positive cells ± 95% confidence limits (CL) of the total number of cells counted in each specific condition and analyzed by χ^2^ test. The mean of TUNEL-positive cells per field from 10–24 fields of 3 tumor sections from each mouse was considered an individual value and data were analyzed by ANOVA. Differences between groups were considered significant when p < 0.05. All the experiments were performed at least twice.

## Supplementary information

Supplementary Information.

## Data Availability

The datasets generated during and/or analysed during the current study are available from the corresponding author on reasonable request.
