# Supplementary material for: Humanin Promotes Tumor Progression in Experimental Triple Negative Breast Cancer
Source: Sci Rep. 2020 May 22;10:8542. doi: 10.1038/s41598-020-65381-7 (PMC7244539; doi:10.1038/s41598-020-65381-7)

HUMANIN PROMOTES TUMOR PROGRESSION IN EXPERIMENTAL TRIPLE NEGATIVE BREAST CANCER

Mariela A. Moreno Ayala[1]*, María Florencia Gottardo[1,2]*, Camila Florencia Zuccato[1]*, Matías L. Pidre[3], Alejandro J. Nicola Candia[1], Antonela S. Asad[1], Mercedes Imsen[1], Víctor Romanowski[3], Aldo Creton[4,5], Marina Isla Larrain[6], Adriana Seilicovich[1,2], Marianela Candolfi[1,a].

1. Instituto de Investigaciones Biomédicas (INBIOMED, UBA-CONICET), Facultad de Medicina, Universidad de Buenos Aires, Argentina.
2. Departamento de Biología Celular e Histología, Facultad de Medicina, Universidad de Buenos Aires. Argentina.
3. Instituto de Biotecnología y Biología Molecular (IBBM, UNLP-CONICET), Facultad de Ciencias Exactas, Universidad Nacional de La Plata, Argentina.
4. Fundación Breast, Calle 7 432/479, B1902, La Plata, Buenos Aires, Argentina.
5. Hospital Italiano, Av. 51, B1900, La Plata, Buenos Aires, Argentina.
6. Centro de Investigaciones Inmunológicas Básicas y Aplicadas (CINIBA), Facultad de Ciencias Médicas, UNLP-CICPBA.

***contributed equally to this work**

[a]Corresponding author:

Dr. Marianela Candolfi
Instituto de Investigaciones Biomédicas (UBA-CONICET)
Facultad de Medicina, Universidad de Buenos Aires
Paraguay 2155, piso 10
Buenos Aires (C1121ABG)
ARGENTINA
mcandolfi@fmed.uba.ar; marucandolfi@gmail.com
Phone/FAX +54-11-59509612

SUPPLEMENTARY MATERIALS

SUPPLEMENTARY FIGURE LEGENDS:

Supplementary Figure 1. Histopathology of primary and metastatic lesions in murine TNBC models. BALB/c mice were s.c. inoculated with $3x10^5$ murine TNBC 4T1 cells. Representative microphotographs show hematoxylin/eosin-stained paraffin sections of primary 4T1 TNBC tumor (**A**) and spontaneous metastases in their lungs (**B**). In the right panel a higher magnification of the area delineated by a black square shows the histological appearance of a metastatic nodule, Arrows indicate mitotic figures.

Supplementary Figure 2. HN expression in human normal and neoplastic mammary gland. Representative microphotographs show HN expression (green) as assessed by immunofluorescence in paraffin sections from mammary gland samples from healthy donors that underwent cosmetic breast surgery (top panels) and tumor biopsies from TNBC patients (lower panels). Nuclei were stained with DAPI (blue).

Supplementary Figure 3. Effect of systemic treatment with chemotherapy and HN on the progression of experimental TNBC. 4T1 TNBC-bearing mice were treated with an injection of liposomal Doxorubicin (DOXO, 100 µg/mouse) at days 15 and 21 post-tumor inoculation. Mice received 6 injections of HN (10 µg/mouse) administered every other day starting at day 15. Tumor size was measured with caliper 3 times a week. The lines indicate tumor size of each mouse at every time point assessed (n=8) in each of the four experimental groups.

SUPPLEMENTARY MATERIALS AND METHODS

HN detection by Flow cytometry

Cells were harvested, fixed with PFA 0.01% and then permeabilized with 0.1% saponin (MP Biomedicals, Inc., Solon, OH) for 10 min. Next, cells were incubated with anti-HN antibody (1 µg/µl, Sigma Aldrich, cat# H2414) in PBS for 1 h at 37 °C. Finally, cells were incubated with anti-rabbit antibody conjugated with FITC (1:50) in PBS for 1 h at 37 °C.

The cells were washed, resuspended in PBS and analyzed by flow cytometry using a FACScan (Becton Dickinson). Data analysis was done with WinMDI 98 software. Cells were incubated with isotype control instead of primary antibody to determine the cut-off for HN fluorescence.

Immunohistochemistry

The presence of HN in 4T1 and MDA-MB-231 TNBC tumor cells was assessed by immunofluorescence. Briefly, PFA-fixed cells in coverslips were blocked with 10% goat serum and incubated with anti-HN antibody (1:100, Sigma, cat# H2414) for 1h, washed, and incubated with anti-rabbit IgG antibody conjugated with fluorescein (1:50, Vector Laboratories Inc.) for 1h. Cells were stained with 4,6-diamidino-2-phenylindoledihydrochloride (DAPI) and mounted with Vectashield.

HN expression was assessed in tumors and lungs from 4T1 TNBC-bearing mice and in human non-neoplastic breast and TNBC sections by immunocytochemistry. Paraffin sections (4 μm) were deparaffinized in xylene and rehydrated in graded ethanol.

4T1 tumor and lung sections were washed in PBS and incubated with 5% skim milk, 0.01% Triton X-100, and then treated with avidin–biotin blocking solution (Vector Laboratories). After incubation with anti-HN antibody (1:100, Sigma, cat# H2414), sections were incubated with biotinylated anti-goat Ig (Vector Laboratories). Endogenous peroxidase activity was blocked by treatment with 0.3% H_2O_2 in methanol for 30 min. Then, sections were incubated with the ABC Vectastain kit following manufacturer´s instruction, and 3,3′-diaminobenzidine–H_2O_2 (DAB Substrate kit; Vector Laboratories) was used as peroxidase substrate. Sections were counterstained with hematoxylin. Microphotographs were obtained in an Axiophot microscope (Carl Zeiss, Jena, Germany) equipped with an Olympus CCD model DP73 digital camera using the Cell Sens Standard program.

Human breast sections were blocked with 10% goat serum and incubated with anti-HN antibody (1:100, NOVUS, cat# NB100-56877SS) for 24h, washed, and incubated with anti-rabbit IgG antibody conjugated with fluorescein (1:200, Vector Laboratories Inc.) for

1h. Then, were stained with 4,6-diamidino-2-phenylindoledihydrochloride (DAPI) and mounted with Vectashield. HN expression was quantified in 19-20 random images from each sample acquired keeping the exposure time constant for all fields. Then, the images were analyzed using the ImageJ software (Version: 1.52t) to evaluate the fluorescence intensity/field. For this analysis, split channels were applied using an automatic gray scale from the green channel. Brightness and contrast were kept constant between the experimental groups. The mean light intensity relative to the total field was obtained. The mean light intensity of background area was obtained for each field. The fluorescence intensity/field was calculated as mean total area - mean light intensity background area. To obtain the fluorescence intensity/field for each sample a mean value was calculated by assessing 20 fields.

BrdU incorporation Assay

$5x10^4$ 4T1 cells were incubated with or without FBS in the presence of 0.5, 1 and 10 μM HN (Genemed Synthesis Inc) for 18 h or in medium with FBS with HN (10 μM) for 2 h before adding liposomal Doxorubicin hydrochloride (DOXO, Dalmonar®, Tuteur, Argentina, 500 nM) for further 72 h. MDA-MB-231 cells were incubated with HN (10 μM) in medium with FBS for 2 h before adding DOXO (250 nM) for further 72 h. Proliferation was assessed by BrDU incorporation. Briefly, 10 μM BrdU labeling solution was added to the media for the last 4 h of cell culture and BrdU incorporation into cellular DNA strands was assessed by ELISA following manufacturer's instructions (ROCHE, Cat# 11647229001). Absorbance was measured in an ELISA reader at 450 nm.

Viability assay (MTT)

4T1 and MDA-MB-231 cells were incubated with different doses of DOXO in medium with FBS for 72 h and cell viability was assessed by MTT assay. Briefly, cells were washed with Krebs solution (0.9% NaCl, 1.15% KCl, 1.22% $CaCl_2$, 2.1% KH_2PO_4, 3.8% $MgSO_4$, 2.6% $NaHCO_3$) and incubated with 3-(4,5-dimethylthiazol-2-yl)-2,5-

diphenyltetrazol (MTT, Molecular Probes) for 4 h. Following the addition of 6N HCl solution, absorbance was determined in a plate reader at 595nm.

Microscopic detection of DNA fragmentation by TUNEL

The apoptotic response of 4T1 cells was evaluated after incubation with HN (10 μM) in medium without FBS for 24 h. In other experiments, 4T1 cells were incubated in medium with FBS with HN (10μM) for 2 h before the addition of TNF-α (50 ng/ml) or DOXO (500nM) for further 24 h. Apoptotic cells were detected using the TUNEL method. Briefly, cells were fixed with cold 4% PFA for 20 min and then permeabilized by microwave irradiation (370W for 5 min) in 10 mM citrate buffer (pH 6). DNA strand breaks were labelled with digoxigenin-dUTP using TdT (0.18 U/μl) according to the manufacturer's protocol (Roche Molecular Biochemicals). After incubation with 10% donkey serum in PBS for 60 min, cells were incubated for 1 h with fluorescein conjugated anti-digoxygenin antibody (1:10) to detect incorporation of nucleotides into the 3'-OH end of damaged DNA. Control cells were incubated without TdT. Nuclei were stained with DAPI as described above and coverslips were mounted in anti-fade mounting medium. Apoptotic cells were counted under fluorescence light microscope.

Apoptotic cells were also assessed in 4T1 tumor sections by the TUNEL method. Sections were deparaffinized and irradiated in citrate buffer, followed by permeabilization in 0.1% Triton X-100 in 0.1% sodium citrate for 5 min at 4°C. Non-specific labeling was prevented by incubating the preparations with blocking solution (5% blocking reagent; Roche Molecular Biochemicals, in 150mM NaCl and 100mM maleic acid, pH 7.5) for 30 min at room temperature. DNA strand breaks were labelled with digoxigenin-deoxyuridine triphosphate using terminal deoxynucleotidyl transferase (0.5 U/μl). Slides were mounted with Vectashield. Apoptotic cells/field were counted under fluorescence light microscope. The mean of TUNEL-positive cells per field from 10-24 fields of 3 tumor sections from each mouse was considered an individual value.

TNBC tumor model

BALB/c female mice were inoculated s.c. into the flank with $3x10^5$ 4T1 cells. When tumors were macroscopic (day 15), mice were treated with an i.p. injection of DOXO (100 μg/mouse), that was repeated at day 21. Mice were also treated with 6 injections of HN (10 μg/mouse), administered every other day, starting on day 15. Tumor measurement was performed 3 times per week using a caliper. Tumor volume was calculated with the following formula: $(width^2xlong)/2$. After the treatment, mice were euthanized under deep anaesthesia by terminal perfusion with Tyrode solution (132 mM NaCl, 1.8 mM $CaCl_2$, 0.32 mM NaH_2PO_4, 5.56 mM glucose, 11.6 mM $NaHCO_3$, and 2.68 mM KCl) followed by Bouin fixative solution [71% picric acid (saturated), 24% formaldehyde (40%), 5% glacial acetic acid]. Lungs were dissected and spontaneous metastases were counted under binocular microscope. Tumors were dissected and processed for TUNEL staining.

Quantitative Real Time PCR

Real Time PCR was performed using a StepOne™ Real-TimePCR System (Applied Biosystems, Carlsbad, CA) and Power SyberGreen PCR Master Mix (Applied Biosystems), as per manufacturer's instructions. All reactions were performed in triplicate. Negative controls included amplification of RNA (without reverse transcription) and water. PCR product specificity was verifed by a melting curve analysis. Linearity of real-time RT-PCR signaling was determined with wide-range serial dilutions of reference cDNA and linear correlations were found between the amount of cDNA and the Ct. Gene expression was normalized to the endogenous reference gene mCYP by the ΔΔCt method using Step-One Software (Applied Biosystems), and expressed as fold-changes relative to the control group. This method was validated in our experiments by confirming that amplification efficiencies of target gene and housekeeping gene were similar. The PCR products were visualized in an agarose gel to confirm fragment size.

SUPPLEMENTARY FIGURES

Supplementary Figure 1. Histopathology of primary and metastatic lesions in murine TNBC models

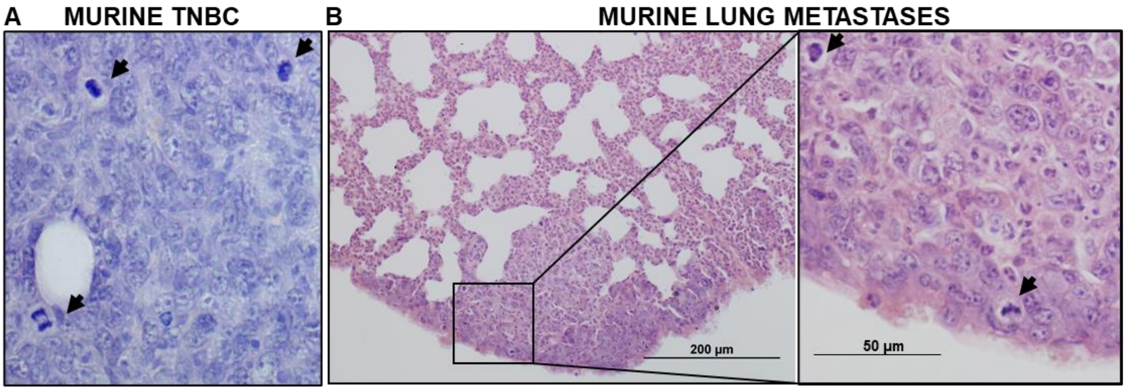

Supplementary Figure 2. HN expression in human normal and neoplastic mammary gland

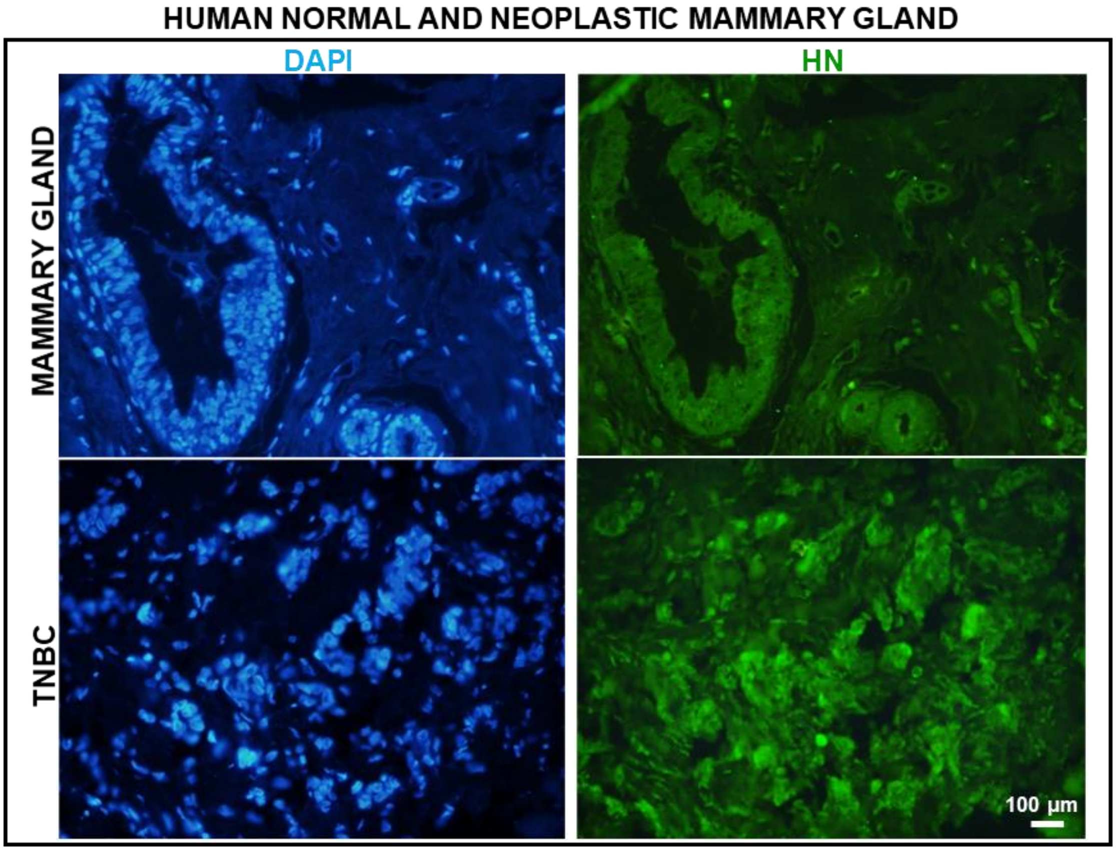

Supplementary Figure 3. Effect of systemic treatment with chemotherapy and HN on the progression of experimental TNBC

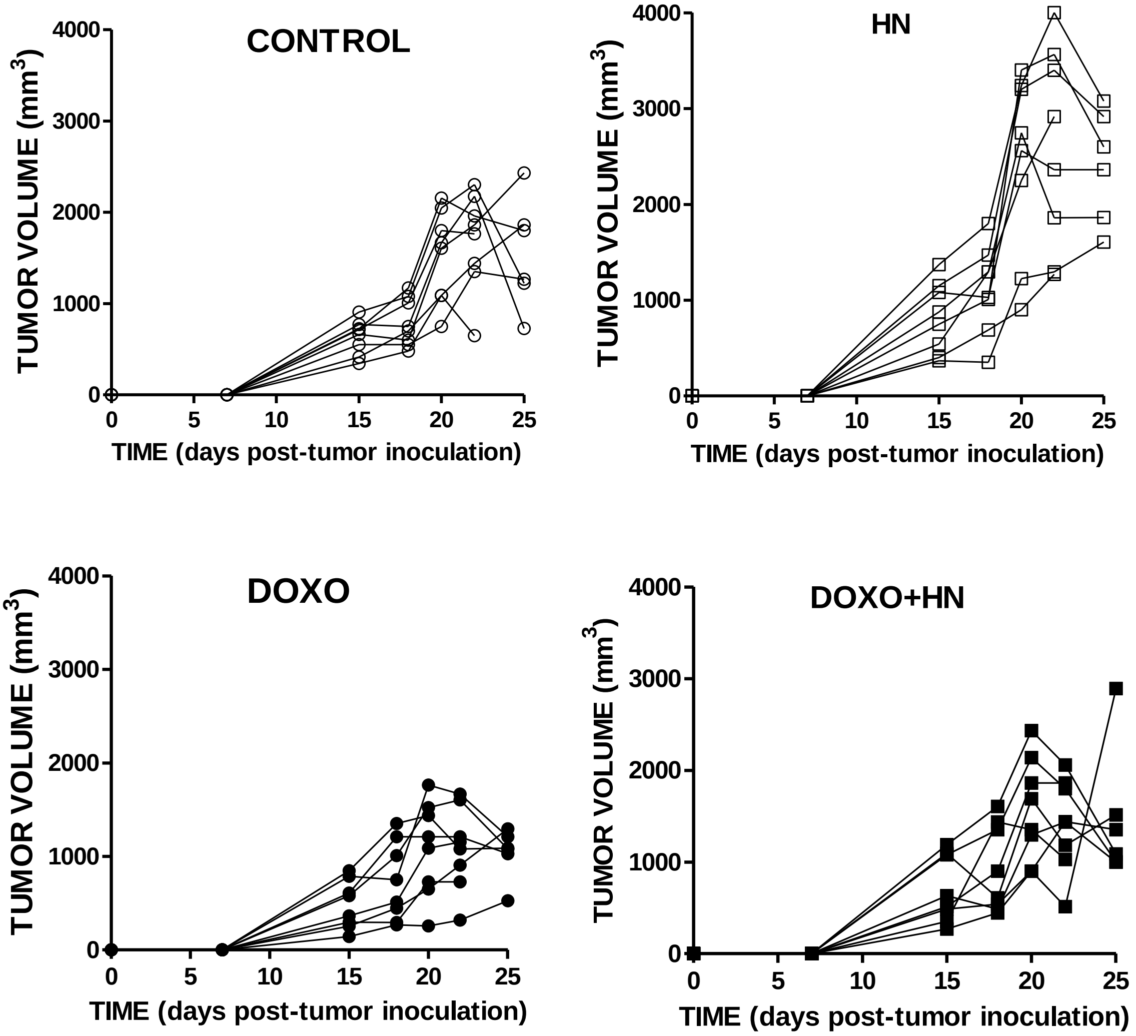

Supplement: Supplementary file 1 — Supplementary Information. [file 41598_2020_65381_MOESM1_ESM.pdf]
